# Sharing a Preliminary Experience of an Effective Online Teaching Strategy for Physiology Practicals

**DOI:** 10.7759/cureus.19388

**Published:** 2021-11-09

**Authors:** Anindita Mahanta, L Sundareswaran, Krishnan S, Abhishek Sinha, Naveen P, Manasi Bhattacharjee

**Affiliations:** 1 Physiology, All India Institute of Medical Sciences, Guwahati, IND

**Keywords:** covid-19, practical skills, first year medical students, online teaching, physiology practical

## Abstract

To counter the challenge posed by the suspension of face-to-face classes due to COVID-19 imposed restrictions, a plan was formulated to conduct practical physiology classes for first-year undergraduate medical students at a newly established medical college in India. The students were provided with study materials before the scheduled class along with an assignment based on it. The class was taken on an online platform, with live practical demonstration on a full-body mannequin. This was followed by discussion in small groups. This strategy actively engaged both teachers and students and provided an effective model for imparting practical skills on an online platform.

## Introduction

The coronavirus disease 2019 (COVID-19) pandemic has affected every aspect of our lives. Education is one such majorly affected area, as implementation of COVID-19 protocol has resulted in the discontinuation of traditional classroom teaching. Although the academic fraternity has braced up to the challenge by adopting various online modes of education, the overall process of teaching and learning has been substantially slowed down. Moreover, some courses cannot be taught entirely on an online mode. This is particularly true in the case of MBBS curriculum where students learn and acquire skills by observing, practicing, and interacting with their teachers, seniors, and patients.

The curriculum for the MBBS course is traditionally divided into pre-clinical, para-clinical, and clinical phases, with varying degrees of horizontal and vertical integration among various phases. Physiology, a pre-clinical discipline, lays the foundation for clinical medicine and is taught in the first year of medical school primarily with the help of didactic lectures and hands-on practical training in laboratories. Laboratory activities play a central role in physiology as it helps reinforce the understanding of theoretical concepts and development of clinical and procedural skills [1,2]. It provides peer support and teaches teamwork, all of which are important for collaborative learning. The hands-on experience of laboratories makes the process of learning more engaging and motivating and helps in the development of a scientific temperament. Although the compulsory shift to online teaching-learning methods due to the pandemic has provided us with an opportunity to integrate technology with the teaching-learning methodologies and make learning student-centered, it is also a great setback for the traditional way of teaching and learning.

Though imparting theoretical knowledge on an online platform could be effective, imparting procedural skills of practicals is particularly challenging in a virtual setting [3,4]. Medical schools have adopted various strategies to address the latter issue. One of the most common modes adopted by pre-clinical subjects is sharing of pre-recorded videos on practical procedures with student groups [5,6]. The students are expected to learn by viewing these videos followed by a doubt-clearing session and discussion. This entire experience is passive and is mostly dependent on the student’s readiness to learn. The teacher in this case is solely transferring the information. The passive nature of this strategy usually does not promote effective learning as the teaching-learning experience becomes more enriching when both the learner and teacher are equally involved [7].

To address the aforementioned shortcomings, a plan was developed to engage students and teachers equally to enable a wholesome teaching-learning experience in spite of its virtual nature. The plan was similar to a flipped classroom model wherein study material is provided to the students prior to class and the class duration time is utilized for active discussion and promotion of critical thinking skills [8]. This strategy was applied mostly for the practicals involving clinical examination of various body systems. The hematology practicals had already been taken on offline mode, and based on their experience, the faculty members collectively devised this teaching plan to ensure the continuity of practical classes.

## Technical report

**Figure 1:**
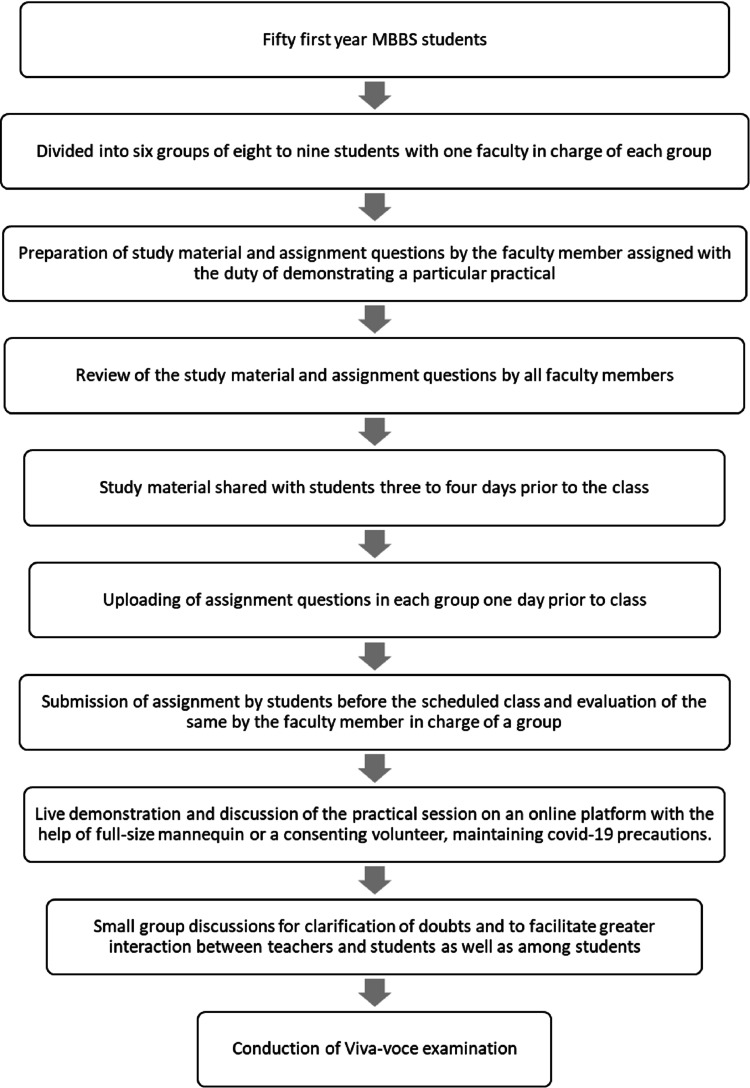
Flowchart showing the steps of the teaching plan described in the report

The students were divided into six groups consisting of eight to nine students (Figure 1). Each group was assigned to a faculty member. Online small group discussions could be conveniently conducted in these groups. In addition, the online platform used had features of sharing and uploading study material and related assignments. Also, proctored written tests and viva voce could be conducted.

A PowerPoint presentation containing the salient points for a particular human physiology practical demonstration was prepared by the faculty member entrusted with the responsibility of taking the practical demonstration. All faculty members reviewed the study material in the presentation and provided their inputs. It was then shared with the students at least three to four days prior to the scheduled class. The study material highlighted the general principles, precautions, and procedure of the practical. The format for reporting the findings was also provided. One day prior to the practical class, an assignment containing a maximum of 10 questions related to the study material was shared by the individual faculty members in their assigned student group. These questions were prepared after considerable brainstorming among faculty members, and the key answers to the same were also finalized to maintain uniformity and avoid bias during discussion sessions with the students. Students were instructed to go through the study material and submit the assignment via an online platform in their respective groups before the practical class. This helped in identification of areas that required more emphasis during the discussion. This was followed by live demonstration of the practical on an online platform. The entire practical was demonstrated using full-body mannequin. For demonstration of some skills, the help of a consenting volunteer was sought. Proper COVID precautions were maintained throughout the class. During the practical demonstration, students were encouraged to clarify their doubts online by using the various features of the online platform such as hand raise and chatbox. This helped recreate the atmosphere similar to offline practical class by promoting the continuous interaction between students and teachers and facilitated an active learning process as opposed to the passive learning that occurs while watching a pre-recorded video. Furthermore, the availability of the study material (PowerPoint presentation) and study questions prior to the scheduled class provided a platform for active discussion among students and teachers. To further facilitate students’ understanding and to promote consolidation of the concepts related to the particular practical demonstration class, the topic was also discussed in the pre-assigned small groups. The whole process of teaching learning adopted for the practical demonstration classes for clinical examination of the various systems allowed a good amount of communication and interaction among the students and teachers to overcome the shortcoming of suspension of face-to-face offline classes. A practical viva voce examination was also conducted in small groups, which allowed one-on-one interaction between faculty members and students. This helped the teachers to identify and grade the level of understanding of the students with regard to the clinical examination skills and also assisted the students’ clarification of minor doubts and correction of errors in clinical examination skills.

## Discussion

The current pandemic situation has highlighted the need for effective integration of technology into the traditional medical curriculum. While the importance of face-to-face didactic lectures and small group discussions cannot be undermined, we also have to keep in mind the potential risks for disease transmission that may occur in the implementation of these methodologies [9]. The utilization of various technologies such as videoconferencing [10], e-learning platforms [11], and simulations with high-fidelity mannequins [5] can ensure continuation of the teaching-learning process while avoiding the risk of contact and disease transmission. While describing the implementation of educational innovations in a medical school in a developing country during the COVID-19 pandemic, the authors have described their experiences as “challenging, exciting, fulfilling, and rewarding” while acknowledging the need for further increase our knowledge in terms of optimal blending of technology into the traditional medical curriculum [5]. Similar emotions were also experienced by the team implementing the teaching strategy discussed in this report.

The use of the flipped classroom as a teaching-learning tool has become increasingly popular in recent times. A recent meta-analysis [12] has shown that the flipped classroom approach promoted student learning, is favored by students compared to the traditional classrooms, and can be made more effective by the use of quizzes. The teaching plan described in this technical report used a methodology that is similar to the flipped classroom approach, wherein students are primed with study material before they come to class so that the class time may be used to assess students’ understanding and stimulate their critical thinking abilities. The main highlight of this teaching strategy, however, was the live demonstration and discussion of a practical session. As mentioned earlier, this ensured considerable similarity to a face-to-face classroom situation.

Students’ expectations from an online learning strategy include an active online participation by the teacher and a good online platform for large group meetings [8]. Also, interactions among students were also perceived to have a positive impact on student learning [8]. The strategy described in the technical report provided sufficient online participation by the faculty members as well as a suitable platform for both large and small group meetings, which encouraged interactions between both teachers and students as well as among students.

The above strategy to impart online practical demonstration classes in human physiology was developed by the faculty members of the Department of Physiology of a newly established institute in India in response to the government restrictions imposed during the COVID-19 pandemic. The faculty members faced several challenges while developing this teaching plan, which have been presented in Table 1.

**Table 1:** Challenges faced while developing the teaching plan and their solutions

Sl. no.	Challenges faced	Strategy devised to overcome it
1	Internet connectivity: for the smooth conduct of the online classes, an uninterrupted internet connection with good speed is required.	The institute’s wi-fi connection was used. In addition, three to four mobile wi-fi networks were kept as backup.
2	Availability of volunteer: owing to the ongoing COVID-19 pandemic and implementation of COVID-19 protocols, it was difficult to find subjects for demonstration of practical skills.	The issue was resolved to some extent by using a full-body mannequin for demonstration of certain procedural techniques. For others, the help of a consenting volunteer was sought and all COVID-19 protocols were maintained.
3	Selection of a suitable online platform: This was a challenge because of the presence of audio and/or video lag in some platforms and restriction on the maximum number of participants allowed in free versions.	Trial sessions with students and faculty members were held on several platforms, and the decision regarding the platform to be used was taken based on the feedback received from the students and faculty members.
4	Maintaining student attention: Holding students’ attention and ensuring their involvement during the class is a challenge on any online platform.	Several opportunities were provided for student-teacher interaction prior to, during, and after the class in the form of PowerPoint presentations, assignments, live demonstration on an online platform, and small group discussions to ensure active participation. The live demonstration especially helped replicate a face-to-face session.
5	Requirement of equipment and financial implications: Conducting the online live demonstration required a full-body mannequin and materials for recording (camera, tripod stand, etc.).	Approval for funds required to purchase the required items was sought from the institute authorities. Live demonstration was done in a smart classroom with camera and internet facilities. In addition, mobile cameras were also used to record from different angles to provide a clear view.
6.	Time constraint: The entire teaching plan had to be devised within a very short span of time due to sudden imposition of lockdown, resulting in suspension of offline classes.	This was made possible by a dedicated group of faculty members who devoted a significant amount of time to develop the strategy, study materials, and assignments followed by intradepartmental review and revision. Also, the active participation of the students, including their feedback helped in improvising the plan on-the-go.

By adapting to the new normal, the students and faculty members stood up to the challenge of the COVID-19 pandemic. Each faculty member was engaged in the task of creation of study material, which was shared in a faculty WhatsApp group for comments and suggestions. This process of peer review allowed for fine-tuning of the study material, making it more comprehensive and efficient. Our experience with the conduct of practical physiology classes on an online platform has demonstrated that even with the challenges of COVID-19 lockdown and social distancing, we can continue the process of teaching learning in an effective manner, with active participation of both teachers and students.

Study limitations

However, like any other online educational activity, this model is also limited by the pre-requisite of an uninterrupted internet connection along with a smartphone or other devices. Also, it is not possible to effectively demonstrate all practical skills on a normal full-body mannequin. The availability of a high-fidelity simulation mannequin would have made the practical demonstration more interactive and life-like.

The way forward

This model can serve as a prototype/template, which can be adopted by other institutions with use of basic and limited online facilities. Also, there is scope to further build on this model to adapt it for use in broader areas of teaching and learning. Optimal utilization of online teaching methods and integration into the curriculum make the process of teaching and learning an active process, with inputs from both teachers and students, which makes it an enriching and fulfilling experience for both.

## Conclusions

Resilience is the ability to adapt to the various changes occurring in our environment. The varied experiences and obstacles that we face in the journey of life shape us as resilient individuals. The current pandemic situation has thrown forward a challenge to the medical education fraternity to incorporate technology in a novel manner to develop a well-structured curriculum, which shall help undergraduate medical students to acquire the knowledge and skills necessary to become clinically competent physicians with a sound scientific background. This model for online teaching of physiology practicals was an optimum blend of technology and teaching strategies. The zeal of the faculty members to maximize learning among students and the live nature of the sessions were the most important factors in accomplishing this feat. We hope that this model shall provide a framework for further improvisation and show a path to adopt a flexible approach to develop a dynamic and resilient education system.

## References

[1] Dohn NB, Fago A, Overgaard J, Madsen PT, Malte H (2016). Students' motivation toward laboratory work in physiology teaching. Adv Physiol Educ.

[2] Hofstein A, Lunetta VN (2004). The laboratory in science education: foundations for the twenty first century. Sci Ed.

[3] Davis CP, Pinedo T (2021). The challenges of teaching anatomy and physiology laboratory online in the time of COVID-19. J Microbiol Biol Educ.

[4] Zhang X, Al-Mekhled D, Choate J (2021). Are virtual physiology laboratories effective for student learning? A systematic review. Adv Physiol Educ.

[5] Owolabi J, Bekele A (2021). Implementation of innovative educational technologies in teaching of anatomy and basic medical sciences during the COVID-19 pandemic in a developing country: the COVID-19 silver lining?. Adv Med Educ Pract.

[6] Wakode SL, Wakode NS (2018). Enhancement of student centred learning using video based practical demonstration in first year medical undergraduates. J Clin Diagn Res.

[7] Salmi L (2013). Student experiences on interaction in an online learning environment as part of a blended learning implementation: what is essential?. IADIS Int Conf Learn.

[8] Ramnanan CJ, Pound LD (2017). Advances in medical education and practice: student perceptions of the flipped classroom. Adv Med Educ Pract.

[9] Liang ZC, Ooi SB, Wang W (2020). Pandemics and their impact on medical training: lessons from Singapore. Acad Med.

[10] Lamba P (2011). Teleconferencing in medical education: a useful tool. Australas Med J.

[11] Kim S (2006). The future of E-learning in medical education: current trend and future opportunity. J Educ Eval Health Prof.

[12] Hew KF, Lo CK (2018). Flipped classroom improves student learning in health professions education: a meta-analysis. BMC Med Educ.

